# (3,6-Dichloro­pyridin-2-yl)(3,5-dimethyl-1*H*-pyrazol-1-yl)methanone

**DOI:** 10.1107/S1600536808015006

**Published:** 2008-06-07

**Authors:** Yue Zhuang, Shan-Shan Zhang, Xian-Hong Yin, Kai Zhao, Cui-Wu Lin

**Affiliations:** a Department of Chemistry, Guangxi University for Nationalities, Nanning 530004, People’s Republic of China; b Department of Chemistry, Guangxi University, Nanning 530006, People’s Republic of China; c College of Chemistry and Chemical Engineering, Guangxi University, Nanning 530006, People’s Republic of China

## Abstract

In the crystal structure of the title compound, C_11_H_9_Cl_2_N_3_O, mol­ecules are held together by short inter­molecular Cl⋯Cl contacts [3.319 (1) Å] and C—H⋯N hydrogen bonds, forming two-dimensional networks parallel to (01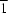).

## Related literature

For related literature, see: Mann *et al.* (1992 ▶); Perevalov *et al.* (2001 ▶).
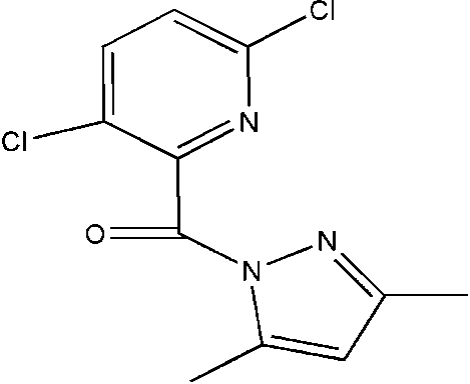


## Experimental

### 

#### Crystal data

C_11_H_9_Cl_2_N_3_O
*M* *_r_* = 270.11
Triclinic, 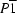

*a* = 7.3440 (10) Å
*b* = 8.7981 (12) Å
*c* = 9.6490 (14) Å
α = 75.554 (2)°
β = 89.627 (3)°
γ = 86.819 (2)°
*V* = 602.79 (15) Å^3^
*Z* = 2
Mo *K*α radiation
μ = 0.52 mm^−1^
*T* = 298 (2) K
0.50 × 0.49 × 0.48 mm

#### Data collection

Bruker SMART CCD area-detector diffractometer
Absorption correction: multi-scan (*SADABS*; Sheldrick, 1996 ▶) *T*_min_ = 0.780, *T*_max_ = 0.787
3145 measured reflections
2102 independent reflections
1543 reflections with *I* > 2σ(*I*)
*R*_int_ = 0.038

#### Refinement

*R*[*F* ^2^ > 2σ(*F* ^2^)] = 0.044
*wR*(*F* ^2^) = 0.126
*S* = 1.04
2101 reflections
154 parameters
H-atom parameters constrained
Δρ_max_ = 0.22 e Å^−3^
Δρ_min_ = −0.27 e Å^−3^

### 

Data collection: *SMART* (Siemens, 1996 ▶); cell refinement: *SAINT* (Siemens, 1996 ▶); data reduction: *SAINT*; program(s) used to solve structure: *SHELXS97* (Sheldrick, 2008 ▶); program(s) used to refine structure: *SHELXL97* (Sheldrick, 2008 ▶); molecular graphics: *SHELXTL* (Sheldrick, 2008 ▶); software used to prepare material for publication: *SHELXTL*.

## Supplementary Material

Crystal structure: contains datablocks I, global. DOI: 10.1107/S1600536808015006/bg2182sup1.cif

Structure factors: contains datablocks I. DOI: 10.1107/S1600536808015006/bg2182Isup2.hkl

Additional supplementary materials:  crystallographic information; 3D view; checkCIF report

## Figures and Tables

**Table 1:** Hydrogen-bond geometry (Å, °)

*D*—H⋯*A*	*D*—H	H⋯*A*	*D*⋯*A*	*D*—H⋯*A*
C11—H11*C*⋯N1^i^	0.96	2.56	3.514 (4)	174

Symmetry code: (i) 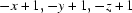.

**Fig. 1.**
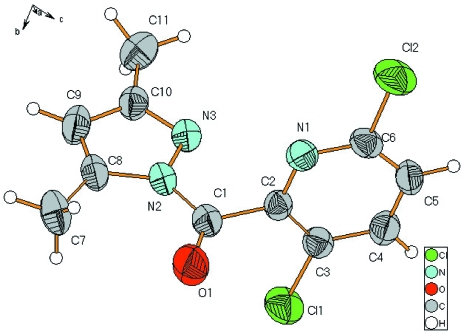
The structure of the title compound (I) showing 50% probability displacement ellipsoids and the atom-numbering scheme.

**Fig. 2.**
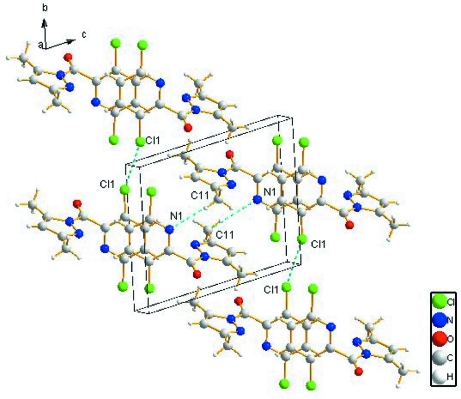
Crystal packing of (I) showing the short contacts interactions as dashed lines.

## supplementary crystallographic information

### Comment

The chemical and pharmacological properties of pyrazoles have been investigated extensively, owing to their chelating ability with metal ions and their potentially beneficial chemical and biological activities (Mann *et al.*, 1992, Perevalov *et al.*, 2001). As part of our studies on the synthesis and characterization of these compounds, we report here the synthesis and crystal structure of (3,6-dichloropyridin-2-yl)(3,5-dimethyl-1*H*-pyrazol-1-yl)methanone.

The crystal structure of the monomeric title compound is built up by C_11_H_9_Cl_2_N_3_O molecules (Fig.1), linked by intermolecular C—H···N hydrogen bonds along [100] (Table 1) and by Cl···Cl short contacts along the [011] direction (Cl1···Cl1^ii^: 3.319 (1)Å, (ii): 2-x, 1-y, 1-z) , forming a two-dimensional network parallel to (011).

The short C=O bond length (1.199 (3)Å) indicates that the molecule is in a keto form (Fig.1). The two rings are nearly perpendicular to each other (dihedral angle: 82.319 (84) °), and this fact helps in minimizing steric effects between them. Finally, there is an intermolecular π—π contact between pyridine groups with and intercentroid distance of 3.40 (1) Å, which contributes to the stability of the crystal packing.

### Experimental

A solution of 3,6-dichloropicolinoyl chloride (10 mmol) in 50 ml toluene was added to a solution of 3,5-dimethyl-1*H*-pyrazole (10 mmol) in 10 ml toluene. The reaction mixture was refluxed for 1 h with stirring then the resulting white precipitate was obtained by filtration, washed several times with ethanol and dried *in vacuo* (yield 90%). Elemental analysis calculate: Elemental analysis: found: C, 48.89; H, 3.33; N, 15.56; calc. for C_11_H_9_Cl_2_N_3_O: C, 48.99; H, 3.23; N, 15.46.

### Refinement

Data collection: 2102 independent reflections but 2101 in Refinement. H atoms on C atoms were positoned geometrically and refined using a riding model with C—H = 0.96Å and *U*_iso_(H) = 1.2*U*_eq_(C).

### Crystal data

**Table:**

C_11_H_9_Cl_2_N_3_O	*Z* = 2
*M**_r_* = 270.11	*F*_000_ = 276
Triclinic, *P*1	*D*_x_ = 1.488 Mg m^−^^3^
Hall symbol: -P 1	Mo *K*α radiation λ = 0.71073 Å
*a* = 7.3440 (10) Å	Cell parameters from 1363 reflections
*b* = 8.7981 (12) Å	θ = 2.2–27.4º
*c* = 9.6490 (14) Å	µ = 0.52 mm^−^^1^
α = 75.554 (2)º	*T* = 298 (2) K
β = 89.627 (3)º	Block, colourless
γ = 86.819 (2)º	0.50 × 0.49 × 0.48 mm
*V* = 602.79 (15) Å^3^	

### Data collection

**Table:**

Bruker SMART CCD area-detector diffractometer	2102 independent reflections
Radiation source: fine-focus sealed tube	1543 reflections with *I* > 2σ(*I*)
Monochromator: graphite	*R*_int_ = 0.038
*T* = 298(2) K	θ_max_ = 25.0º
φ and ω scans	θ_min_ = 2.2º
Absorption correction: multi-scan(SADABS; Sheldrick, 1996)	*h* = −8→8
*T*_min_ = 0.780, *T*_max_ = 0.787	*k* = −9→10
3145 measured reflections	*l* = −9→11

### Refinement

**Table:**

Refinement on *F*^2^	Secondary atom site location: difference Fourier map
Least-squares matrix: full	Hydrogen site location: inferred from neighbouring sites
*R*[*F*^2^ > 2σ(*F*^2^)] = 0.044	H-atom parameters constrained
*wR*(*F*^2^) = 0.126	*w* = 1/[σ^2^(*F*_o_^2^) + (0.0468*P*)^2^ + 0.2168*P*] where *P* = (*F*_o_^2^ + 2*F*_c_^2^)/3
*S* = 1.04	(Δ/σ)_max_ < 0.001
2101 reflections	Δρ_max_ = 0.22 e Å^−^^3^
154 parameters	Δρ_min_ = −0.27 e Å^−^^3^
Primary atom site location: structure-invariant direct methods	Extinction correction: none

### Special details

**Table:**

Geometry. All e.s.d.'s (except the e.s.d. in the dihedral angle between two l.s. planes) are estimated using the full covariance matrix. The cell e.s.d.'s are taken into account individually in the estimation of e.s.d.'s in distances, angles and torsion angles; correlations between e.s.d.'s in cell parameters are only used when they are defined by crystal symmetry. An approximate (isotropic) treatment of cell e.s.d.'s is used for estimating e.s.d.'s involving l.s. planes.
Refinement. Refinement of *F*^2^ against ALL reflections. The weighted *R*-factor *wR* and goodness of fit *S* are based on *F*^2^, conventional *R*-factors *R* are based on *F*, with *F* set to zero for negative *F*^2^. The threshold expression of *F*^2^ > σ(*F*^2^) is used only for calculating *R*-factors(gt) *etc*. and is not relevant to the choice of reflections for refinement. *R*-factors based on *F*^2^ are statistically about twice as large as those based on *F*, and *R*- factors based on ALL data will be even larger.

### Fractional atomic coordinates and isotropic or equivalent isotropic displacement parameters (Å^2^)

**Table:**

	*x*	*y*	*z*	*U*_iso_*/*U*_eq_	
Cl1	0.60627 (11)	0.85128 (9)	0.94981 (8)	0.0634 (3)	
Cl2	0.90422 (12)	0.19393 (9)	0.92556 (10)	0.0712 (3)	
N1	0.8160 (3)	0.4899 (3)	0.8173 (2)	0.0434 (5)	
N2	0.6305 (3)	0.7559 (2)	0.5846 (2)	0.0415 (5)	
N3	0.4844 (3)	0.6627 (2)	0.6266 (2)	0.0421 (5)	
O1	0.8838 (3)	0.8463 (3)	0.6636 (2)	0.0712 (7)	
C1	0.7630 (4)	0.7572 (3)	0.6853 (3)	0.0440 (6)	
C2	0.7517 (3)	0.6330 (3)	0.8231 (2)	0.0368 (6)	
C3	0.6925 (3)	0.6653 (3)	0.9493 (3)	0.0407 (6)	
C4	0.6996 (4)	0.5468 (3)	1.0739 (3)	0.0469 (7)	
H4	0.6596	0.5662	1.1598	0.056*	
C5	0.7666 (4)	0.4004 (3)	1.0686 (3)	0.0488 (7)	
H5	0.7746	0.3181	1.1508	0.059*	
C6	0.8220 (4)	0.3782 (3)	0.9384 (3)	0.0445 (7)	
C7	0.7566 (5)	0.9440 (4)	0.3653 (3)	0.0670 (9)	
H7A	0.7593	1.0332	0.4056	0.101*	
H7B	0.7259	0.9789	0.2654	0.101*	
H7C	0.8743	0.8890	0.3764	0.101*	
C8	0.6167 (4)	0.8362 (3)	0.4409 (3)	0.0483 (7)	
C9	0.4615 (4)	0.7924 (3)	0.3939 (3)	0.0548 (8)	
H9	0.4145	0.8254	0.3013	0.066*	
C10	0.3819 (4)	0.6865 (3)	0.5110 (3)	0.0444 (6)	
C11	0.2080 (4)	0.6066 (4)	0.5140 (3)	0.0622 (8)	
H11A	0.1074	0.6782	0.5223	0.093*	
H11B	0.2091	0.5166	0.5944	0.093*	
H11C	0.1953	0.5733	0.4272	0.093*	

### Atomic displacement parameters (Å^2^)

**Table:**

	*U*^11^	*U*^22^	*U*^33^	*U*^12^	*U*^13^	*U*^23^
Cl1	0.0743 (6)	0.0459 (4)	0.0697 (5)	0.0120 (4)	0.0039 (4)	−0.0172 (4)
Cl2	0.0775 (6)	0.0408 (4)	0.0948 (7)	0.0118 (4)	−0.0144 (5)	−0.0188 (4)
N1	0.0444 (13)	0.0417 (13)	0.0441 (13)	0.0003 (10)	−0.0022 (10)	−0.0110 (10)
N2	0.0448 (13)	0.0369 (12)	0.0379 (12)	−0.0010 (10)	0.0017 (10)	−0.0006 (9)
N3	0.0440 (13)	0.0414 (12)	0.0388 (12)	−0.0010 (10)	0.0006 (10)	−0.0062 (9)
O1	0.0706 (15)	0.0756 (16)	0.0611 (14)	−0.0326 (13)	−0.0013 (11)	0.0012 (11)
C1	0.0466 (16)	0.0413 (15)	0.0422 (15)	−0.0033 (13)	0.0020 (12)	−0.0066 (12)
C2	0.0338 (13)	0.0387 (14)	0.0360 (13)	−0.0027 (11)	−0.0032 (10)	−0.0058 (11)
C3	0.0382 (14)	0.0390 (14)	0.0438 (15)	0.0000 (12)	−0.0031 (11)	−0.0084 (12)
C4	0.0507 (17)	0.0510 (17)	0.0380 (15)	−0.0034 (13)	−0.0011 (12)	−0.0088 (12)
C5	0.0527 (18)	0.0462 (17)	0.0406 (15)	−0.0082 (13)	−0.0079 (13)	0.0036 (12)
C6	0.0415 (15)	0.0343 (14)	0.0559 (17)	0.0001 (11)	−0.0102 (12)	−0.0083 (12)
C7	0.079 (2)	0.062 (2)	0.0488 (18)	−0.0075 (18)	0.0099 (16)	0.0091 (15)
C8	0.0604 (19)	0.0408 (15)	0.0373 (15)	0.0049 (13)	0.0037 (13)	0.0009 (12)
C9	0.064 (2)	0.0595 (19)	0.0359 (15)	0.0079 (16)	−0.0081 (14)	−0.0047 (13)
C10	0.0488 (16)	0.0445 (15)	0.0382 (14)	0.0057 (12)	−0.0024 (12)	−0.0090 (12)
C11	0.0570 (19)	0.076 (2)	0.0561 (19)	−0.0005 (17)	−0.0078 (15)	−0.0219 (16)

### Geometric parameters (Å, °)

**Table:**

Cl1—C3	1.722 (3)	C5—C6	1.375 (4)
Cl2—C6	1.732 (3)	C5—H5	0.9300
N1—C6	1.325 (3)	C7—C8	1.496 (4)
N1—C2	1.334 (3)	C7—H7A	0.9600
N2—C1	1.382 (3)	C7—H7B	0.9600
N2—N3	1.383 (3)	C7—H7C	0.9600
N2—C8	1.392 (3)	C8—C9	1.340 (4)
N3—C10	1.316 (3)	C9—C10	1.419 (4)
O1—C1	1.199 (3)	C9—H9	0.9300
C1—C2	1.502 (3)	C10—C11	1.488 (4)
C2—C3	1.380 (3)	C11—H11A	0.9600
C3—C4	1.380 (4)	C11—H11B	0.9600
C4—C5	1.366 (4)	C11—H11C	0.9600
C4—H4	0.9300		
			
C6—N1—C2	117.4 (2)	C5—C6—Cl2	119.8 (2)
C1—N2—N3	118.3 (2)	C8—C7—H7A	109.5
C1—N2—C8	130.2 (2)	C8—C7—H7B	109.5
N3—N2—C8	111.5 (2)	H7A—C7—H7B	109.5
C10—N3—N2	104.7 (2)	C8—C7—H7C	109.5
O1—C1—N2	123.1 (2)	H7A—C7—H7C	109.5
O1—C1—C2	121.5 (2)	H7B—C7—H7C	109.5
N2—C1—C2	115.3 (2)	C9—C8—N2	105.3 (2)
N1—C2—C3	122.1 (2)	C9—C8—C7	131.3 (3)
N1—C2—C1	114.8 (2)	N2—C8—C7	123.4 (3)
C3—C2—C1	123.0 (2)	C8—C9—C10	107.6 (2)
C4—C3—C2	119.4 (3)	C8—C9—H9	126.2
C4—C3—Cl1	120.5 (2)	C10—C9—H9	126.2
C2—C3—Cl1	120.03 (19)	N3—C10—C9	110.9 (2)
C5—C4—C3	118.7 (3)	N3—C10—C11	120.8 (2)
C5—C4—H4	120.6	C9—C10—C11	128.3 (2)
C3—C4—H4	120.6	C10—C11—H11A	109.5
C4—C5—C6	118.1 (2)	C10—C11—H11B	109.5
C4—C5—H5	121.0	H11A—C11—H11B	109.5
C6—C5—H5	121.0	C10—C11—H11C	109.5
N1—C6—C5	124.3 (3)	H11A—C11—H11C	109.5
N1—C6—Cl2	115.9 (2)	H11B—C11—H11C	109.5
			
C1—N2—N3—C10	179.3 (2)	Cl1—C3—C4—C5	179.1 (2)
C8—N2—N3—C10	−0.6 (3)	C3—C4—C5—C6	−0.7 (4)
N3—N2—C1—O1	−171.8 (3)	C2—N1—C6—C5	0.0 (4)
C8—N2—C1—O1	8.1 (5)	C2—N1—C6—Cl2	179.37 (18)
N3—N2—C1—C2	11.2 (3)	C4—C5—C6—N1	0.5 (4)
C8—N2—C1—C2	−169.0 (2)	C4—C5—C6—Cl2	−178.9 (2)
C6—N1—C2—C3	−0.2 (4)	C1—N2—C8—C9	−179.9 (3)
C6—N1—C2—C1	175.1 (2)	N3—N2—C8—C9	0.0 (3)
O1—C1—C2—N1	−98.9 (3)	C1—N2—C8—C7	1.1 (5)
N2—C1—C2—N1	78.2 (3)	N3—N2—C8—C7	−179.1 (3)
O1—C1—C2—C3	76.4 (4)	N2—C8—C9—C10	0.5 (3)
N2—C1—C2—C3	−106.5 (3)	C7—C8—C9—C10	179.5 (3)
N1—C2—C3—C4	0.0 (4)	N2—N3—C10—C9	0.9 (3)
C1—C2—C3—C4	−174.9 (2)	N2—N3—C10—C11	−179.3 (2)
N1—C2—C3—Cl1	−178.62 (19)	C8—C9—C10—N3	−0.9 (3)
C1—C2—C3—Cl1	6.4 (3)	C8—C9—C10—C11	179.3 (3)
C2—C3—C4—C5	0.4 (4)		

### Hydrogen-bond geometry (Å, °)

**Table:**

*D*—H···*A*	*D*—H	H···*A*	*D*···*A*	*D*—H···*A*
C11—H11C···N1^i^	0.96	2.56	3.514 (4)	174

Symmetry codes: (i) −*x*+1, −*y*+1, −*z*+1.
